# Scalp and genital ulcerations as cutaneous toxicities of amivantamab and lazertinib combination therapy

**DOI:** 10.1016/j.jdcr.2026.05.036

**Published:** 2026-05-22

**Authors:** Joseph Lozenski, Olivia Graham, Urmi Khanna

**Affiliations:** a Division of Dermatology, Department of Internal Medicine, University of Kansas Medical Center and School of Medicine, Kansas City, Kansas; b Department of Dermatology, Dartmouth Hitchcock Medical Center and Geisel School of Medicine at Dartmouth, Lebanon, New Hampshire

**Keywords:** amivantamab, biologic toxicity, chemotherapy, cutaneous toxicity, EGFR-Met inhibitor, erosive pustular dermatosis–like reaction, scalp and genital ulceration

## Introduction

Amivantamab is a bispecific epidermal growth factor receptor–hepatocyte growth factor receptor (EGFR-MET) inhibitor indicated for patients with advanced non-small cell lung cancer (NSCLC) with EGFR Exon 20 insertions.^1^ EGFR signaling is essential for keratinocyte proliferation, skin maturation, hair follicle development, and maintenance of follicular immune privilege.^2^ MET is a transmembrane receptor that plays important roles in vascular permeability and wound healing.^3^

Dysregulated EGFR signaling drives oncogenesis in NSCLC, leading to widespread use of targeted EGFR tyrosine kinase inhibitors (EGFR TKIs) in advanced disease; however, tumor resistance frequently develops through MET amplification. Amivantamab targets both EGFR and MET signaling, resulting in superior progression-free survival when combined with standard chemotherapy (carboplatin and pemetrexed) compared with standard chemotherapy alone (11.4 months versus 6.7 months, HR = 0.38).^1^ In addition, amivantamab with lazertinib (EGFR TKI) has also demonstrated superior survival compared to osimertinib alone (EGFR TKI) (HR = 0.75).^4^

Despite its oncologic benefit, amivantamab is associated with a high incidence of cutaneous adverse events, affecting approximately 90% patients. These include conventional EGFR-associated toxicities of acneiform eruption and paronychia. Retrospective studies report acneiform eruptions in nearly 70% of patients, xerosis in approximately 50%, paronychia in 30%, and hair changes in 13% to 44%. Approximately 10% to 35% of patients on amivantamab therapy required treatment interruption or discontinuation due to cutaneous toxicity.^5^

In addition to the cutaneous adverse effects seen with EGFR TKIs, amivantamab has been associated with severe, refractory ulcerative eruptions involving the scalp and genital regions. Scalp involvement initially resembles pityriasis amiantacea, with crusts overlying erosions, and has also been described as an erosive pustular dermatosis (EPD)–like eruption.^5^, ^6^, ^7^, ^8^ The severity and scalp-predominant nature of these reactions suggest a mechanism distinct from that of EGFR TKIs alone. It is hypothesized that the dual EGFR-MET blockade of amivantamab may induce more pronounced folliculocentric injury compared to EGFR TKIs. MET inhibition is known to increase vascular permeability and may further compromise vascular supply to hair follicles.^9^ Together, impaired hair follicle repair, wound healing, and vascular supply via MET inhibition may exacerbate EGFR inhibition-related injuries predisposing patients to the severe rashes and ulcerations observed with amivantamb.^3^

## Case presentation

A 62-year-old female with NSCLC and brain metastases presented to the dermatology clinic with painful paronychia, scalp ulcerations, and genital lesions approximately 12 months after initiation of amivantamab plus lazertinib, following brain radiation.

**Fig 1:**
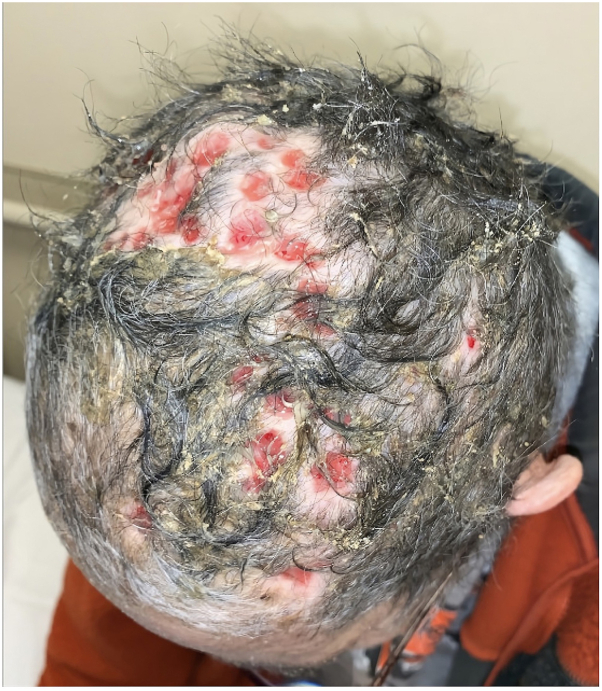
Clinical presentation of cutaneous toxicity following initiation of amivantamab plus lazertinib. Scalp showing punched-out, well-defined ulcers with a clean base and exuberant granulation tissue, resembling a pseudopyogenic granuloma. The adjacent scalp demonstrates matting of hair due to purulent discharge and overlying crusting.

Approximately 8 months after the initiation of therapy, the patient developed pustules and thick scaling on the scalp, resembling pityriasis amiantacea. This scalp eruption rapidly progressed, and by month 12, multiple deep ulcers with punched-out margins and exuberant granulation tissue were observed (Fig 1).

Paronychia developed after 10 months of therapy and progressed to involve all fingers and toes by month 12. Examination of the genital region revealed erythematous papules and plaques consistent with pseudopyogenic granulomas overlying erosions and ulcerations.

**Fig 2:**
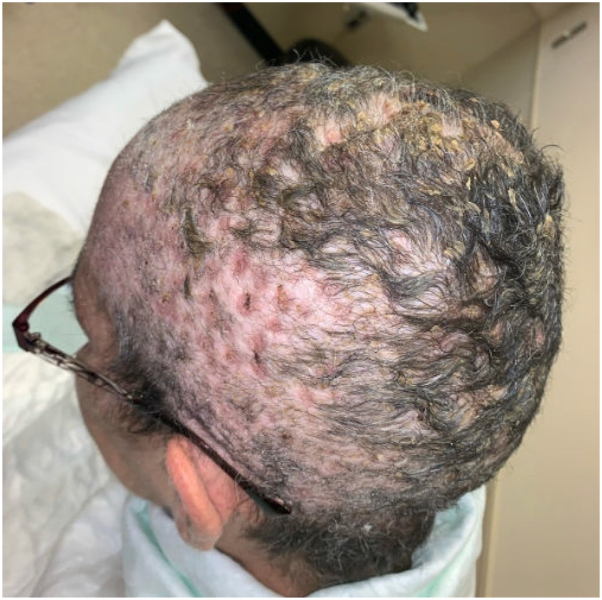
Clinical improvement of scalp ulcers 3 months after discontinuation of amivantamab, while continuing lazertinib therapy. The patient’s scalp ulcers were also managed with silver dressings, tacrolimus ointment, and 4% chlorhexidine gluconate cleanser.

Paronychia was managed with mupirocin and moisturization, while the EDP-like eruption was treated with silver dressings. After 12 months of persistent cutaneous toxicities, amivantamab was held indefinitely, while lazertinib was continued. These cutaneous toxicities only improved after amivantamab cessation (Fig 2). The genital pseudopyogenic granulomas with ulcerations improved with amivantamab discontinuation, tacrolimus ointment, and 4% chlorhexidine gluconate cleanser.

## Discussion

This case demonstrates the persistent and severe nature of amivantamab-associated cutaneous toxicities, with meaningful clinical improvement occurring only after amivantamab cessation. EPD-like scalp ulceration has a later onset and more persistent course than other EGFR toxicities, typically occurring 4 to 13 months after initiation, and may develop even if other skin findings stabilize or improve.^6^ EPD-like eruptions are becoming more recognized, with 1 recent multicenter retrospective review describing 31% of patients with ≥grade 2 dermatologic adverse events developed scalp ulcerations, while 7% experienced genital ulcerations.^8^ This case further supports amivantamb as the primary driver of EPD-like eruptions, as improvement only occurred after cessation of amivantamab cessation, regardless of whether conventional EGFR TKI therapy is continued.^5^, ^6^, ^7^, ^8^, ^9^

Amivantamab-induced genital ulcers are increasingly recognized and have been reported to be refractory to management with systemic and topical steroids.^6^, ^7^, ^8^ These ulcers share morphologic similarities with scalp ulcers, including well-defined borders and clean bases with increased granulation tissue. Histopathology has shown these scalp ulcers to have a more folliculocentric neutrophil-rich infiltrate; however, florid granulation tissue is present in both scalp and genital ulcers induced by amivantamab, suggesting a possible common pathogenesis.^6^, ^7^, ^8^ Impaired follicular repair due to EGFR-MET inhibition, combined with vascular compromise and inflammation, could contribute to both granulation tissue formation and ulceration. Due to the paucity of reports and difficulty of treating genital toxicity, we urge clinicians to maintain a high index of suspicion when evaluating intertriginous ulcers in patients on amivantamab.

Importantly, there is no evidence for genital ulceration in EGFR TKIs. This unique cutaneous toxicity and higher rate of severe adverse cutaneous events, like EPD-like eruptions, differentiates amivantamab from EGFR TKI dermatologic effects (Table I). These findings suggest that amivantamab’s dual blockade of EGFR and MET signaling may particularly predispose patients to skin and follicocentric injury.

**Table I:** Proposed mechanism of dermatologic toxicity, dermatologic toxicities, and systemic toxicities associated with EGFR TKIs, MET TKIs, and amivantamab

Drug	EGFR TKIs	MET TKIs	EGFR-MET dual inhibitor (Amivantamab)
Proposed mechanism of dermatologic toxicity	Intracellularly binds and inhibits EGFR, disrupting cellular signaling for keratinocyte proliferation and hair follicle development	Intracellularly binds and inhibits MET, disrupting cellular signaling for vascular permeability and wound repair	Extracellularly binds and inhibits both EGFR and MET. Promotes skin injury by disrupting EGFR signaling for keratinocyte proliferation and hair follicle development, which is exacerbated by impaired MET signaling for vascular permeability and wound repair.
Dermatologic toxicities	Skin: acneiform eruptions (30% to 50%), skin fissures/xerosis (20%)	Minimal	Skin: acneiform eruptions (70%), skin fissures/xerosis (15% to 30%), genital ulcerations
	Nails: paronychia (30%)		Nails: paronychia (20% to 50%)
	Hair/scalp: EPD-like eruptions (uncommon), alopecia		Hair/scalp: EPD-like eruptions (30%), alopecia
Other toxicities	Stomatitis, nausea/vomiting	Nausea/vomiting, peripheral edema, hypoalbuminemia	Stomatitis, nausea/vomiting, peripheral edema, hypoalbuminemia, infusion-related reactions, ocular toxicity, interstitial lung disease

*EGFR*, Epidermal growth factor receptor; *EPD*, erosive pustular dermatosis; *NSCLC*, non-small cell lung cancer; *TKI*, tyrosine kinase inhibitor.

Percentages of pertinent toxicities are included in parentheses.^1^, ^2^, ^3^, ^4^, ^5^, ^6^, ^7^, ^8^, ^9^

From a management perspective, wound swabs and/or tissue cultures should be performed to rule out infectious etiologies of ulceration as well as superimposed infection. Prophylactic dermatologic regimens including doxycycline/minocycline, clindamycin, chlorhexidine, and ceramide moisturizer have been shown to reduce ≥grade 2 dermatologic adverse events from 75% to 42%, highlighting the benefit of proactive care.^10^

For refractory cases, systemic steroids, dapsone, and retinoids have been found useful.^6^^,^^8^^,^^9^ If these options fail, therapy cessation may be required. Scalp and genital ulcerations are the leading causes of treatment interruption, highlighting their clinical severity.^8^ However, the true clinical burden may be underestimated, particularly in vulnerable regions such as the scalp and genitalia, where pain and risk of infection are increased.^5^ With the growing use of targeted therapies for multiple molecular pathways, the landscape of cutaneous toxicities is expanding in complexity. Multidisciplinary care, ideally with an early dermatology consultation, is vital for optimizing patient outcomes.

### Declaration of generative AI and AI-assisted technologies in the writing process

During the preparation of this letter, the authors did not use any artificial intelligence tool or service.

## Conflicts of interest

None disclosed.
